# Development of a machine learning-based radiomics signature for estimating breast cancer TME phenotypes and predicting anti-PD-1/PD-L1 immunotherapy response

**DOI:** 10.1186/s13058-024-01776-y

**Published:** 2024-01-29

**Authors:** Xiaorui Han, Yuan Guo, Huifen Ye, Zhihong Chen, Qingru Hu, Xinhua Wei, Zaiyi Liu, Changhong Liang

**Affiliations:** 1 School of Medicine South, China University of Technology, Guangzhou, 510006 China; 2 Department of Radiology, Guangdong Provincial People’s Hospital (Guangdong Academy of Medical Sciences), Southern Medical University, Guangzhou, 510080 China; 3 Guangdong Provincial Key Laboratory of Artificial Intelligence in Medical Image Analysis and Application, Guangzhou, 510080 China; 4 Department of Radiology, Guangzhou First People’s Hospital, School of Medicine, South China University of Technology, Guangzhou, 510180 China; 5 The Second School of Clinical Medicine, Southern Medical University, Guangzhou, 510000 China; 6 Institute of Computing Science and Technology, Guangzhou University, Guangzhou, 510006 China

**Keywords:** Machine learning, Radiomics signature, Breast cancer, Tumor microenvironment

## Abstract

**Backgrounds:**

Since breast cancer patients respond diversely to immunotherapy, there is an urgent need to explore novel biomarkers to precisely predict clinical responses and enhance therapeutic efficacy. The purpose of our present research was to construct and independently validate a biomarker of tumor microenvironment (TME) phenotypes via a machine learning-based radiomics way. The interrelationship between the biomarker, TME phenotypes and recipients’ clinical response was also revealed.

**Methods:**

In this retrospective multi-cohort investigation, five separate cohorts of breast cancer patients were recruited to measure breast cancer TME phenotypes via a radiomics signature, which was constructed and validated by integrating RNA-seq data with DCE-MRI images for predicting immunotherapy response. Initially, we constructed TME phenotypes using RNA-seq of 1089 breast cancer patients in the TCGA database. Then, parallel DCE-MRI images and RNA-seq of 94 breast cancer patients obtained from TCIA were applied to develop a radiomics-based TME phenotypes signature using random forest in machine learning. The repeatability of the radiomics signature was then validated in an internal validation set. Two additional independent external validation sets were analyzed to reassess this signature. The Immune phenotype cohort (*n* = 158) was divided based on CD8 cell infiltration into immune-inflamed and immune-desert phenotypes; these data were utilized to examine the relationship between the immune phenotypes and this signature. Finally, we utilized an Immunotherapy-treated cohort with 77 cases who received anti-PD-1/PD-L1 treatment to evaluate the predictive efficiency of this signature in terms of clinical outcomes.

**Results:**

The TME phenotypes of breast cancer were separated into two heterogeneous clusters: Cluster A, an "immune-inflamed" cluster, containing substantial innate and adaptive immune cell infiltration, and Cluster B, an "immune-desert" cluster, with modest TME cell infiltration. We constructed a radiomics signature for the TME phenotypes ([AUC] = 0.855; 95% CI 0.777–0.932; *p* < 0.05) and verified it in an internal validation set (0.844; 0.606–1; *p* < 0.05). In the known immune phenotypes cohort, the signature can identify either immune-inflamed or immune-desert tumor (0.814; 0.717–0.911; *p* < 0.05). In the Immunotherapy-treated cohort, patients with objective response had higher baseline radiomics scores than those with stable or progressing disease (*p* < 0.05); moreover, the radiomics signature achieved an AUC of 0.784 (0.643–0.926; *p* < 0.05) for predicting immunotherapy response.

**Conclusions:**

Our imaging biomarker, a practicable radiomics signature, is beneficial for predicting the TME phenotypes and clinical response in anti-PD-1/PD-L1-treated breast cancer patients. It is particularly effective in identifying the "immune-desert" phenotype and may aid in its transformation into an "immune-inflamed" phenotype.

**Supplementary Information:**

The online version contains supplementary material available at 10.1186/s13058-024-01776-y.

## Introduction

As indicated in the latest regional report, breast cancer has now surpassed lung cancer in incidence among Chinese women for the first time, making it the most prevalent cancer, accounting for approximately 19.9% of newly diagnosed cases [1]. Minimizing the incidence of recurrence and metastasis five years after surgery, as well as length of life, are essential concerns and obstacles for breast cancer [2]. Present breast cancer treatment have hit a plateau; novel therapeutic strategies are sought [3].In 2019, the U.S. Food and Drug Administration initially authorized the anti-programmed cell death ligand 1 (PD-L1) inhibitor Atezolizumab for the first-line management of PD-L1-positive advanced triple-negative breast cancer, marking a new adventure in immunotherapy for breast cancer [4]. Latest studies, however, have demonstrated that immunotherapies, for example immune checkpoint inhibitors (ICIs), show unsatisfactory efficacy across the board for breast cancer patients [5–7].

The failure to identify patients via the landscape of tumor microenvironment (TME) may be the primary cause of these dismal outcomes. Meanwhile, the complete spectrum of TME phenotypes in breast cancer is currently unexplained. The majority of prior investigations have emphasized on either one or two subtypes of TME breast cancer cells [8–11], which may have resulted in a skewed knowledge of the breast cancer TME. Since TME cells exhibit robust intercellular communication, it is more logical to regard them as a whole.

The emergence of next-generation sequencing has enabled the investigation of the TME. Given the fact that RNA-seq of tumor samples invariably indicates the presence of TME cell types, scientists proposed a variety of genetic expression profile-based measurement of the TME cell abundance in tumor tissue [12–14]. However, conventional TME evaluations often involve the acquisition of tissue biopsies following surgery. It would be beneficial to search out non-invasive techniques for evaluating the TME landscape.

Radiomics is the technique of interpreting medical images and converting them into numerical data [15, 16]. High-dimensional imaging data offer abundant information regarding tumor phenotypes that are governed not merely by the inherent physiological processes of cancer cells, but also by the TME, such as the makeup and infiltrating patterns of tumor-infiltrating immune cells [17]. The purpose of radiomics is to produce image-mediated predictors that act as tools to reveal the association between certain imaging and TME phenotypes and to gain knowledge of breast cancer biology in order to improve therapeutic governance [18, 19]. Radiomics precedes biopsy testing due to its physical noninvasiveness, enabling longitudinal estimation of the tumor and its surrounding milieu, description of spatial variability, as well as disease progression.

Immunotherapy has drastically altered the strategy for treating cancers such as breast cancer [20–22]. Unfortunately, lower than 10% of individuals with specific breast cancer react to the treatment, which is regrettably indicative of the broad variation in patient response to immunotherapy [23, 24]. Therefore, it is necessary to develop strategies for determining which patients are most likely to react to immunotherapy. It is demonstrated that preexisting intratumoral and peritumoral TME cell infiltration associated with anti-programmed cell death protein (PD)-1 and anti-PD-L1 immunotherapy response [25, 26]. There have been characterized three distinct immunological phenotypes: immune-inflamed, immune-excluded, and immune-desert. The immune-inflamed phenotype, referred to as "hot tumors", is defined by tumor cells expressing PD-L1, an enormous prevalence of infiltrating immune cells in the TME, and an increased frequency of tumor-infiltrating lymphocytes (TILs), and these tumors are immunotherapy-receptive [14, 27]. In contrast, immune-excluded and immune-desert phenotypes, referred to as "cold tumors", lack infiltrating inflammatory cells, making immunotherapy typically ineffective, posing a major obstacle to achieving universal effectiveness in immune treatment. It has been demonstrated that transforming "cold tumors" into "hot tumors" can benefit more individuals from immunotherapy [28].

We anticipated that MRI radiomics may capture microstructural variations noninvasively across breast cancer TME phenotypes in order to find new determinants of immunotherapy success. We aim to (i) categorize TME phenotypes in breast cancer and (ii) develop a radiomics signature for the TME phenotypes to determine the clinical outcomes of patients receiving anti-PD-1/PD-L immunotherapy.

## Materials

### Protocol and data sources

**Fig. 1:**
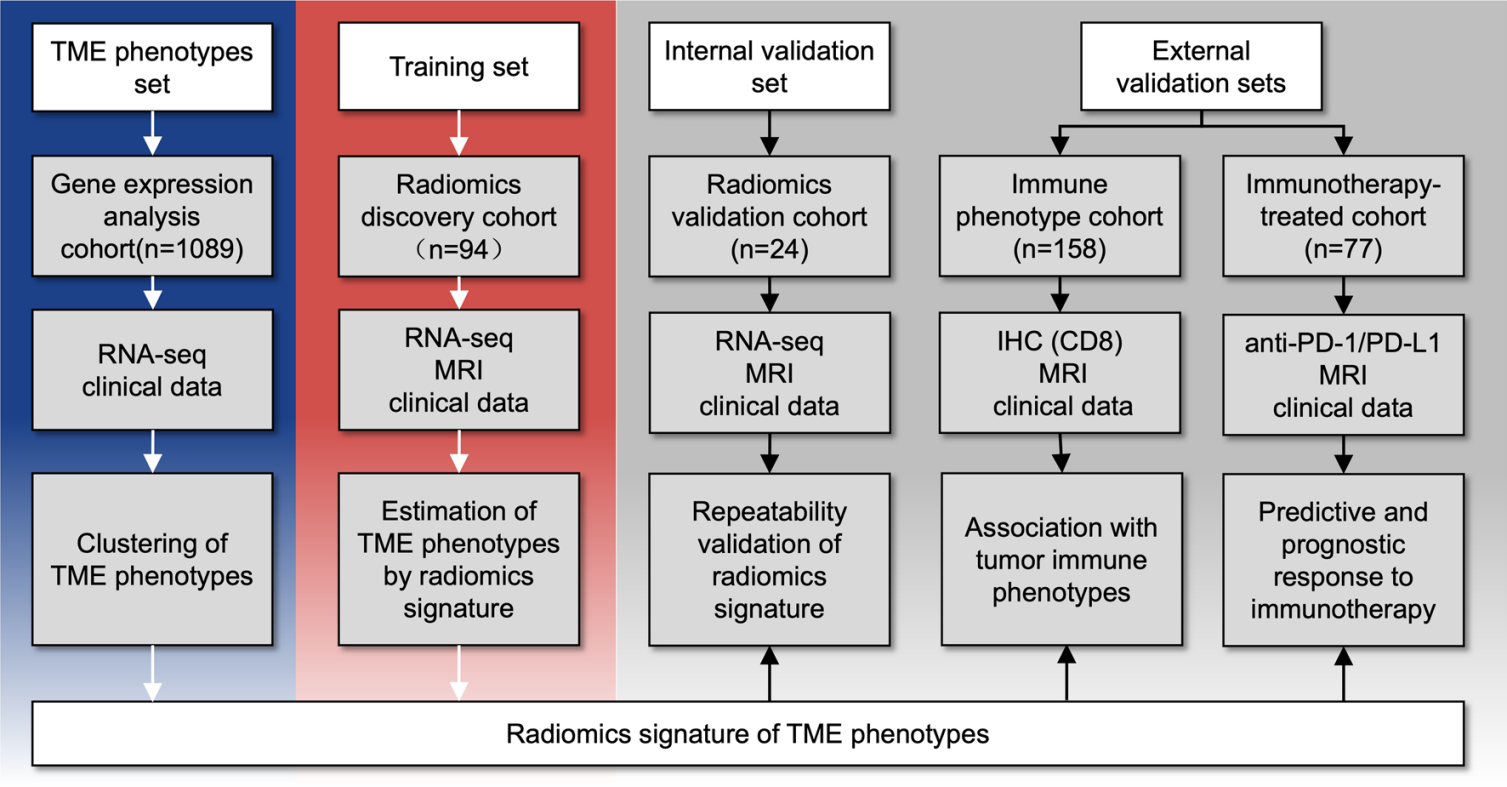
Study design. The Gene expression analysis cohort containing both RNA-seq and clinical data was used to develop TME phenotypes. The Radiomics discovery cohort, regarded as a training set, was used for radiomics signature training of TME phenotypes while the Radiomics validation cohort was for repeatability validation. Data from these two cohort were both derived from the TCIA dataset. The Immune phenotype cohort was divided into immune-inflamed tumors and immune-desert tumors according to the IHC-CD8 outcomes of the enrolled subjects. The Immunotherapy-treated cohort receiving anti-PD-1/PD-L1 therapy was used to predict the prognostic response to immunotherapy. These two external validation sets were recruited from the Guangdong People's Hospital. *TME, Tumor microenvironment; RNA-Seq, RNA sequencing; IHC, Immunohistochemistry; PD-1, programmed cell death protein-1; PD-L1, programmed cell death ligand 1

In this multi-cohort investigation, retrospective genomics and radiomics analysis was carried out on five distinct cohorts of breast cancer patients 18 years or older (Fig. 1). The Cancer Genome Atlas (TCGA) and Cancer Imaging Archive (TCIA) databases provided the information for the Gene expression analysis cohort, the Radiomics discovery cohort, and the Radiomics validation cohort. The Radiomics discovery cohort and the Radiomics validation cohort both originate from the same data collection namely TCGA-BRCA, as the former cohort containing 80% cases and the later one with 20% cases. The Immune phenotype cohort and the Immunotherapy-treated cohort were extracted from the database of the Guangdong Provincial People's Hospital. The Gene expression analysis cohort was comprised of transcriptome data from 1089 patients (the transcriptomic data and relevant clinicopathological variables are included in **Table S1**), for describing the landscape of the breast cancer TME and revealing its heterogeneity. The Radiomics discovery cohort consists of 94 patients whose DCE-MRI and RNA-seq data are available, and all cases permitted prediction of TME phenotypes by radiomics analysis using both RNA-seq data and accompanying MR images.

In conjunction with one internal and two separated external validation sets, the radiomics signature was validated. The Radiomics validation cohort (internal validation set) consisted of 24 patients, and this dataset was utilized to confirm the congruence between the radiomics signature (from the Radiomics discovery cohort) and the TME phenotypes in this independent dataset. The Immune phenotype cohort (external validation set 1) consisted of 158 patients grouped into the two most severe tumor immune phenotypes based on their CD8 immunohistochemistry (IHC) staining findings (CD8 20% was deemed positive) [29, 30]: the immune-inflamed phenotype and the immune-desert phenotype, regardless of the treatment provided. This dataset was employed to reveal the match between the breast cancer radiomics signature and tumor immunophenotype. All 77 breast cancer patients in the Immunotherapy-treated cohort (external validation set 2) underwent anti-PD-1/PD-L1 treatment. This cohort was utilized to identify the relationship between radiomics signature and adherence to the Response Evaluation Criteria for Solid Tumors (RECIST) version 1.1 clinical response. Our research was authorized by the review committee of Guangdong Provincial People's Hospital and conducted in compliance with the Helsinki Declaration's ethical standards. Due to the retroactive nature of the investigation, the committee waived the requirement for informed consent. All trial data were de-identified and made anonymized.

### Calculation of TME cell abundance

Previous study [27] constructed a compendium of breast cancer TME genes associated with specific microenvironment cell subpopulations by modifying two gene signatures, CIBERSORT [31] and MCP-Counter [32]. This compilation includes 324 genes that represented 22 categories of immune cells and 40 genes that represented endothelial cells and fibroblasts. Using single sample gene enrichment analysis (ssGSEA,"GSVA" in R) with expression data, this result was used in our work to compute each cell subpopulation of each sample by measuring their abundance.

### TME phenotypes clustering

To establish the optimal number of stable breast cancer TME subtypes, k-means ("kmeans" in R) clustering, Nbclust testing ("NbClust" in R, index = "all"), and Silhouette analysis ("Silhouette" in R) was used. Prior to clustering, we scaled each sample in order to cluster them according to the compositional pattern of each TME cell type. For heatmap visualization, the data were reordered based on the k-means clustering findings and scaled the original ssGSEA results prior to displaying (“pheatmap in R”).

### Prognostic value of TME phenotypes

A Kaplan–Meier survival curve was plotted using "ggsurvplot" in R to assess the prognostic value of each cell subpopulation in each TME cluster within the total cohort. The optimal cutoff value was determined in each investigation to classify cell abundance as high or low using Youden index.

### MR image analysis and feature extraction

Radiomics features can be affected by equipment and acquisition conditions, therefore we preprocessed the obtained DICOM images using the following steps: (i) Z-score normalization, where we normalize the intensity of all MRI images using the Z-score calculation (Caret package); (ii) Resampling, where we used Simple ITK software to standardize the voxel spacing to 1.0 $\times$ 1.0 $\times$ 1.0 mm^3^.

**Fig. 2:**
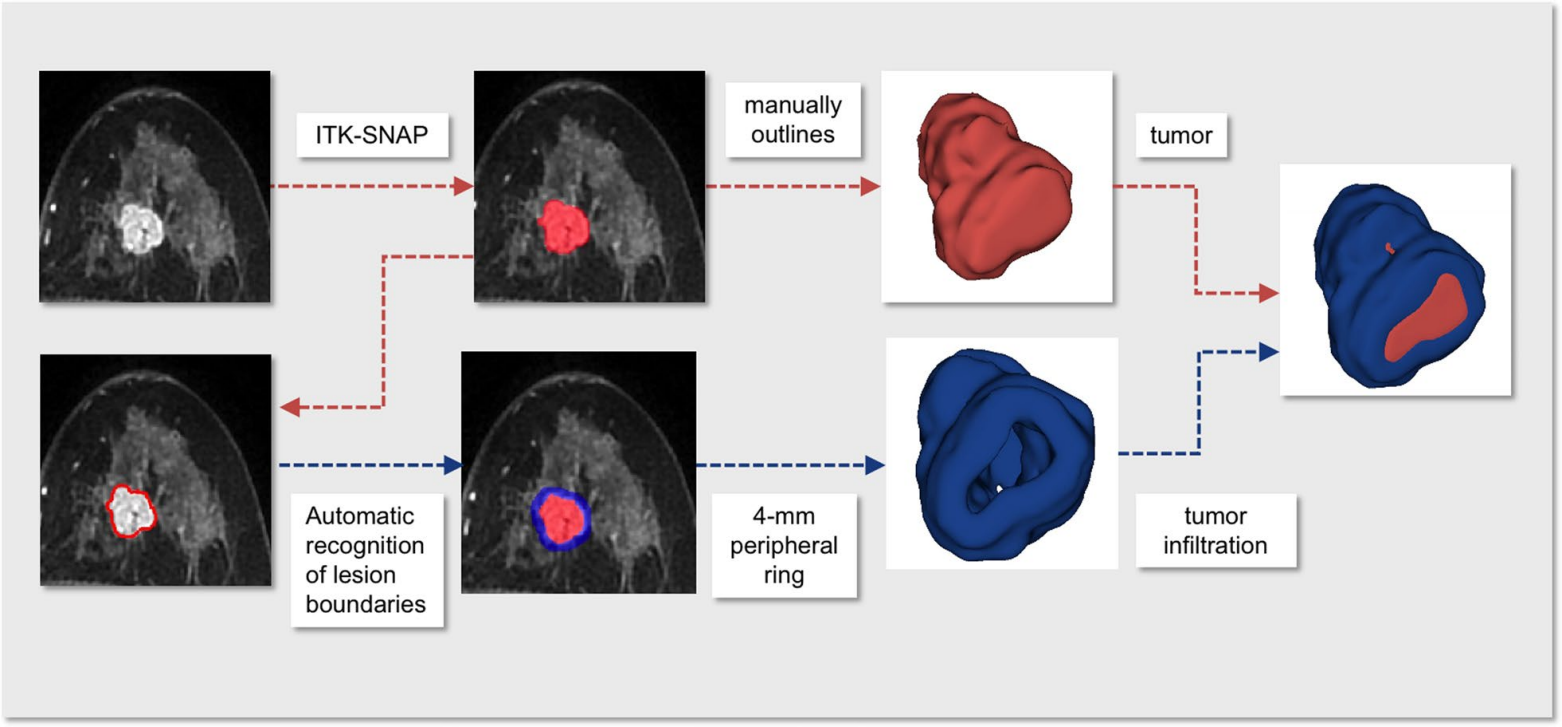
Lesion segmentation. The core tumor area in MRI imaging was measured and outlined manually by ITK-SNAP software in different slices. The boundary of the tumor was automatically identified by Python. A peripheral region was then established inside and outside 2 mm away from the defined boundary, consequently forming a 4-mm thickness ring to represent the extension of the tumor infiltration

The segmentation of the imaging was accomplished semi-automatically. Initially, two radiologists (** and **, with ** and ** years of experience in breast cancer MR diagnosis, respectively) utilized ITK-SNAP software (www.itk-snap.org) to manually draw tumor borders on MRI and establish agreement on tumor outlines; disputes were addressed by consensus. To get quantitative data from the TME, we next utilized Python to automatically augment the lesions depending on the findings of the lesion border identification. On each side of the tumor border (i.e., the outer and inner sides of the border), an expanding and contracting 2 mm peripheral ring was constructed, culminating in a 4-mm ring [33]. Large-size blood arteries, neighboring tissues, and air spaces were all excluded if there was no tumor cell invasion (Fig. 2).

We extracted features for each area of interest, i.e., the intratumoral and peritumoral regions, using Python, pyradiomics. A final collection of 479 quantitative features, including 14 shape features, 90 first-order intensity features, and 375 s-order and higher-order texture features, was extracted. The features extracted from the images were described in detail in Additional file 1: **Tables S2 and S3**.

By evaluating all radiomics features extracted using intra- and inter-class correlation coefficients (ICC), we assess inter- and intra-observer consistency and repeatability. Radiologists ** and ** extracted features from thirty randomly chosen patients. Two weeks later, radiologists ** repeated the identical processes. We selected features with an ICC greater than 0.75 ensured the reliability of extracted features.

### Construction of radiomics signature

In the Radiomics discovery cohort, we created a random forest model to predict the TME phenotypes; fivefold cross-validation picked the best model [34, 35]. This model was applied to the Radiomics validation cohort using the Youden index to determine the best threshold, hence maximizing the overall sensitivity and specificity [34].

Feature significance specifies which features were relevant and enabled the enhancement of a model via feature selection. We calculated feature significance using the random forest algorithm from the scikit-learn package (Python).

### Statistical analysis

The Student-t test, Wilcoxon test, and Kruskal–Wallis test were used to compare continuous variables, and the Shapiro–Wilk test was employed to determine the normality of the distribution prior to comparison [27]. For categorical variables, Fisher's exact test was utilized [27]. The Kaplan–Meier method was used to analyze survival, and the log-rank test was used to compare survival across clusters [27]. ICCs were used to determine inter- and intra-observer consistency in lesion segmentation [36]. A random forest classifier model was used to categorize the TME phenotypes [34, 35]. For the evaluation of model performance, the area under the subject operating characteristic curve (AUC) and other performance assessment metrics (Accuracy, sensitivity, specificity, positive predictive value and negative predictive value) were used to determine if Radiomics score (rad-score) could classify patients into two groups based on the abundance of TME cell infiltration [37]. Spearman's correlation coefficient was utilized to determine the relationship between the 20 most significant radiomics features and the abundance of 24 cell infiltration in TME [38].

In the Immunotherapy-treated cohort, patients were categorized into high or low scoring groups using the median value of the rad-score. The duration of follow-up was calculated from the initiation of immunotherapy. Clinical response was classified as complete response, partial response, stable disease, or progressive disease based on RECIST version 1.1 [39] and assessed at six months; the immunotherapy response for each patient is presented in Additional file 1: Table S4. A two-sided *P* value < 0.05 was considered statistically significant. All statistical analyses were performed using R (Version 3.5.0).

### Role of funding sources

The fundings of the study make contribution to the establishment of breast cancer dataset, but has no roles in study design, data collection, data analysis, data interpretation.

## Results

### Demographic and clinical characteristics

A total of 1442 subjects with a mean age of 57 ± 13 years were included in our present research, among them, 1089 patients (mean age: 58.5 ± 13.2 years) was assigned into the Gene expression analysis cohort, 94 patients (mean age, 54.8 ± 11.2 years) to the Radiomics discovery cohort, 24 patients (mean age, 53 ± 13 years) to the Radiomics validation cohort, 158 patients to the Immune phenotype cohort (mean age, 51.1 ± 11.1 years), and 77 patients to the Immunotherapy-treated cohort (mean age, 50.8 ± 9.1 years). The population characteristics of these five cohorts were exhaustively described in Table 1.

**Table 1:** Characteristics of patients in the Gene expression analysis, Radiogenomics discovery cohort, radiogenomics validation cohort, immune phenotype cohort and Immunotherapy-treated cohort

Variables	Gene expression analysis cohort		Radiogenomics discovery cohort		Radiogenomics validation cohort		Immune phenotype cohort		Immunotherapy-treated cohort	
	n = 1089		n = 94		n = 24		n = 158		N = 77	
	N	%	N	%	N	%	N	%	N	%
Age (years)										
18–60	600	55.1	62	66	18	75	121	76.6	67	87
> 60	489	44.9	32	34	6	25	37	23.4	10	13
Laterality										
Left	567	52.1	50	53.2	10	41.7	89	56.3	51	66.2
Right	522	47.9	44	46.8	14	58.3	69	43.7	26	33.8
Status										
Alive	938	86.1	91	96.8	24	100	NA	NA	NA	NA
Dead	151	13.9	3	3.2	0	0	NA	NA	NA	NA
OS (years)										
≤ 1	168	15.4	6	6.3	1	4.2	NA	NA	NA	NA
> 1 ≤ 3	481	44.2	44	46.3	6	25	NA	NA	NA	NA
> 3 ≤ 5	187	17.2	24	25.3	8	33.3	NA	NA	NA	NA
> 5 years	253	23.2	21	22.1	9	37.5	NA	NA	NA	NA
Pathologic stage										
I	180	16.5	20	21.3	8	33.3	6	3.8	0	0
II	620	57	59	62.7	15	62.5	57	36.1	19	24.7
III	248	22.8	15	16	1	4.2	23	14.5	12	15.6
IV	20	1.8	0	0	0	0	2	1.3	0	0
X	0	0	0	0	0	0	0	0	0	0
NA	21	1.9	0	0	0	0	70	44.3	46	59.7
Estrogen receptor										
Positive	802	73.6	78	83	19	79.2	119	75.3	15	19.5
Negative	237	21.8	16	17	5	20.8	39	24.7	58	75.3
Indeterminate	–	–	0	0	0	0	0	0	0	0
NA	50	4.6	0	0	0	0	0	0	4	5.2
Progesterone receptor										
Positive	693	63.6	72	76.6	15	62.5	100	63.3	15	19.5
Negative	343	31.5	22	23.4	9	37.5	58	36.7	58	75.3
Indeterminate	–	–	0	0	0	0	0	0	0	0
NA	53	4.9	0	0	0	0	0	0	4	5.2
Human epidermal growth factor receptor 2										
Positive	164	15.1	11	11.7	2	8.4	118	74.7	6	7.8
Negative	559	51.3	48	51.1	14	58.3	40	25.3	67	87
Indeterminate	190	17.4	0	0	0	0	0	0	0	0
NA	176	16.2	35	37.2	8	33.3	0	0	4	5.2

NA: not available

### Landscape of TME phenotypes in breast cancer

**Fig. 3:**
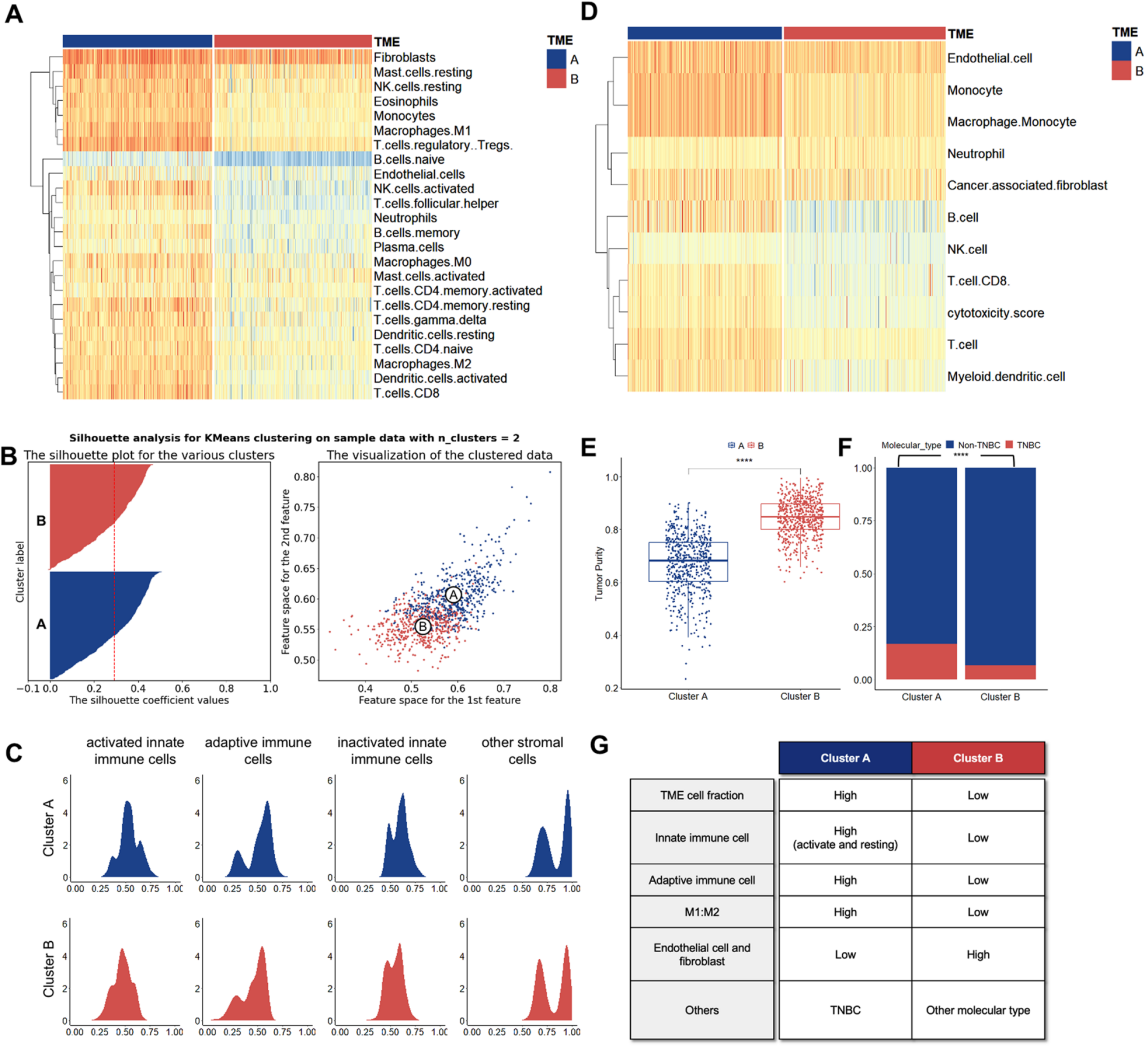
Landscape of TME phenotypes in breast cancer. **A** K-means clustering of breast cancer TME phenotypes demonstrating abundance of 24 TME cell subpopulations measured by ssGSEA. **B** Optimal number of clusters determined by Silhouette analysis. **C** Distribution of characteristic scores of four cell subpopulations in two clusters. **D** MCP-counter assessment of the cellular infiltration level in both phenotypes. **E** Estimated assessment of tumor purity. **F** Distribution of intrinsic subtypes of breast cancer in the clusters. **G** Cellular characteristics of Cluster A and B. * ****, *p* < 0.0001; TNBC, triple-negative breast cancer, M1 and M2, type 1 and type 2 macrophages

The reference TME compendium developed in prior research consisted of 364 genes covering 24 TME cell subpopulations, comprehensively defining the TME phenotypes of breast cancer. This finding was used to measure the abundance of 24 TME cellular subpopulations in each sample (Additional file 1: Table S5). We then ran k-means clustering on the TME phenotypes of breast cancer. All 1089 breast cancer TME phenotypes were categorized into two heterogeneous clusters (Fig. 3A). The ideal and stable number of clusters was two (Fig. 3B** and** Additional file 2: Figure S1). Cluster A, the "immune-inflamed" cluster ("hot tumor"), was shown as relatively large infiltration of immune cells. Cluster B, the "immune-desert" cluster ("cold tumor"), was characterized by poor TME cell infiltration. Furthermore, we characterized the distribution of TME cell subpopulations to determine their relative proportions within clusters. The relative percentage of non-immune and innately inactivated immune cells grew in the Cluster B, while the relative weight of innate and adaptive immune cells increased in the Cluster A. (Fig. 3C). The distribution of clinical variables was not random within the Cluster A and B (Additional file 3: Figure S2).

### Validation of breast cancer TME clusters

To ensure that the two consensus clusters were not influenced by the analysis algorithm, we measured the abundance of cell infiltration for both phenotypes using MCP-counter (Fig. 3D**,** Additional file 1: Table S6) and estimated tumor purity using ESTIMATE (Fig. 3E**)** and ABSOLUTE, Leukocytes Unmethylation for Purity(LUMP), IHC (Additional file 1: Table S7). This was done to validate the stability and robustness of the ssGSEA results. Infiltration of immune cells was much more prevalent in the Cluster A, whereas endothelial cells and fibroblasts were significantly more prevalent in the Cluster B. This result was in line with the findings of the ssGSEA. Additionally, the tumor purity of the Cluster A was much lower than that of the Cluster B. (Fig. 3E). Furthermore, we analyzed the distribution of molecular subtypes of breast cancer in clusters, finding triple-negative breast cancer mostly in the Cluster A and other molecular subtypes in the Cluster B (Fig. 3F, Additional file 3: Figure S2). In conclusion, we revealed that breast tumors exhibit two diverse TME phenotypes. The characteristics of the two clusters were demonstrated in Fig. 3G.

### Prognostic significance of TME cells in breast cancer

**Fig. 4:**
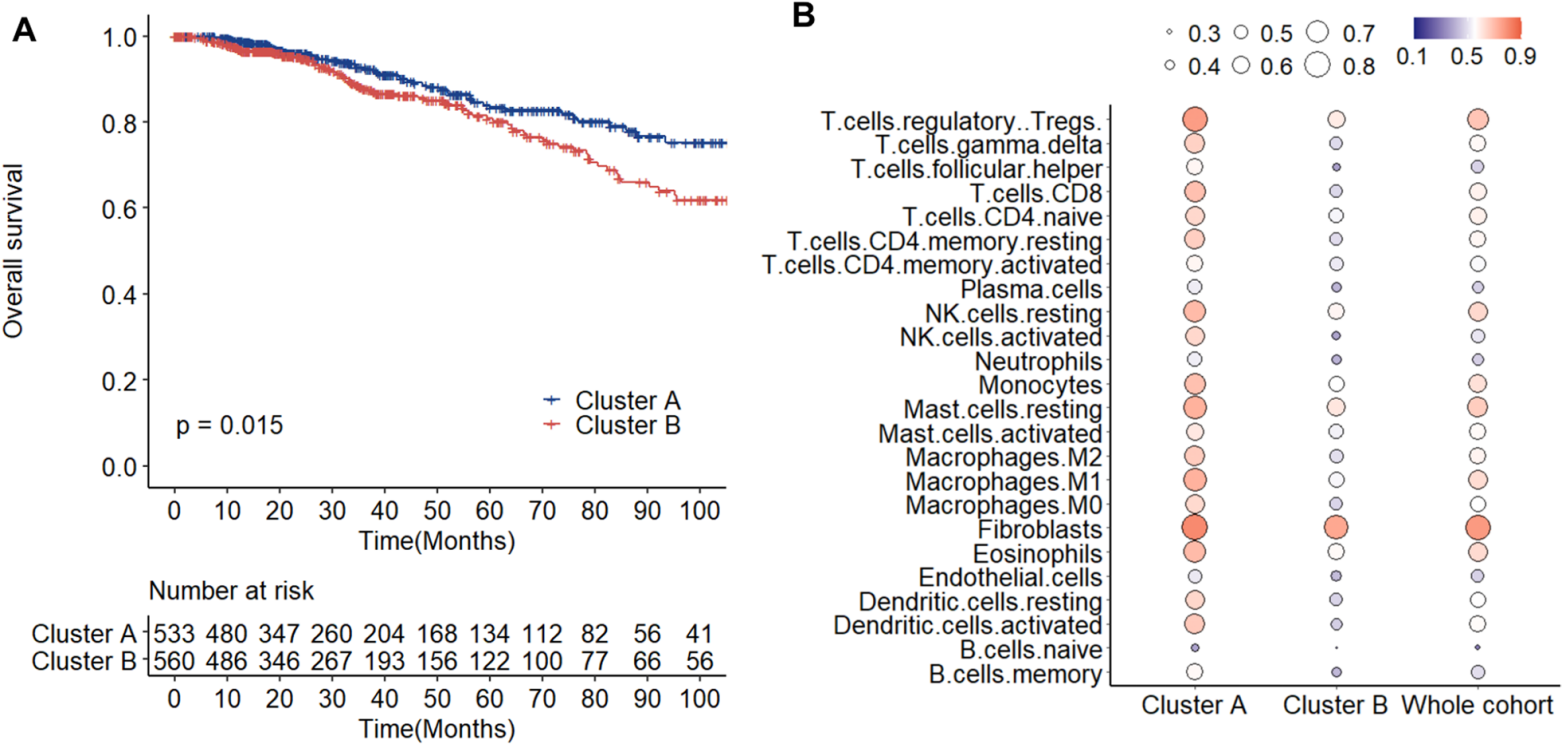
Prognostic significance of TME cells in breast cancer. **A** Kaplan–Meier curves of OS between Cluster A and B. **B** Measurement of the prognostic value of each cell subgroup by a univariate Cox proportional hazards model for OS in the whole cohort, as well as Cluster A and B. Gradually changing color, indicates Hazard radio, and the size of the circles represents − log10 (FDR-P value). Larger circles demonstrate smaller FDR-P values. * TME, tumor microenvironment; OS, overall survival; FDR, false discovery rate

Given the significance of the TME in prognosis of breast cancer, we further studied the prognostic significance of TME clusters. The Cluster A had considerably superior OS (*p* < 0.05) (Fig. 4A). In addition, the prognostic importance of each cell subpopulation was investigated (Fig. 4B). A better prognosis was indicated by a larger degree of immune cell infiltration, including immunosuppressive cell infiltration, in the Cluster A, the Cluster B, and the whole cohort.

### Activation of immune modulators in breast cancer

**Fig. 5:**
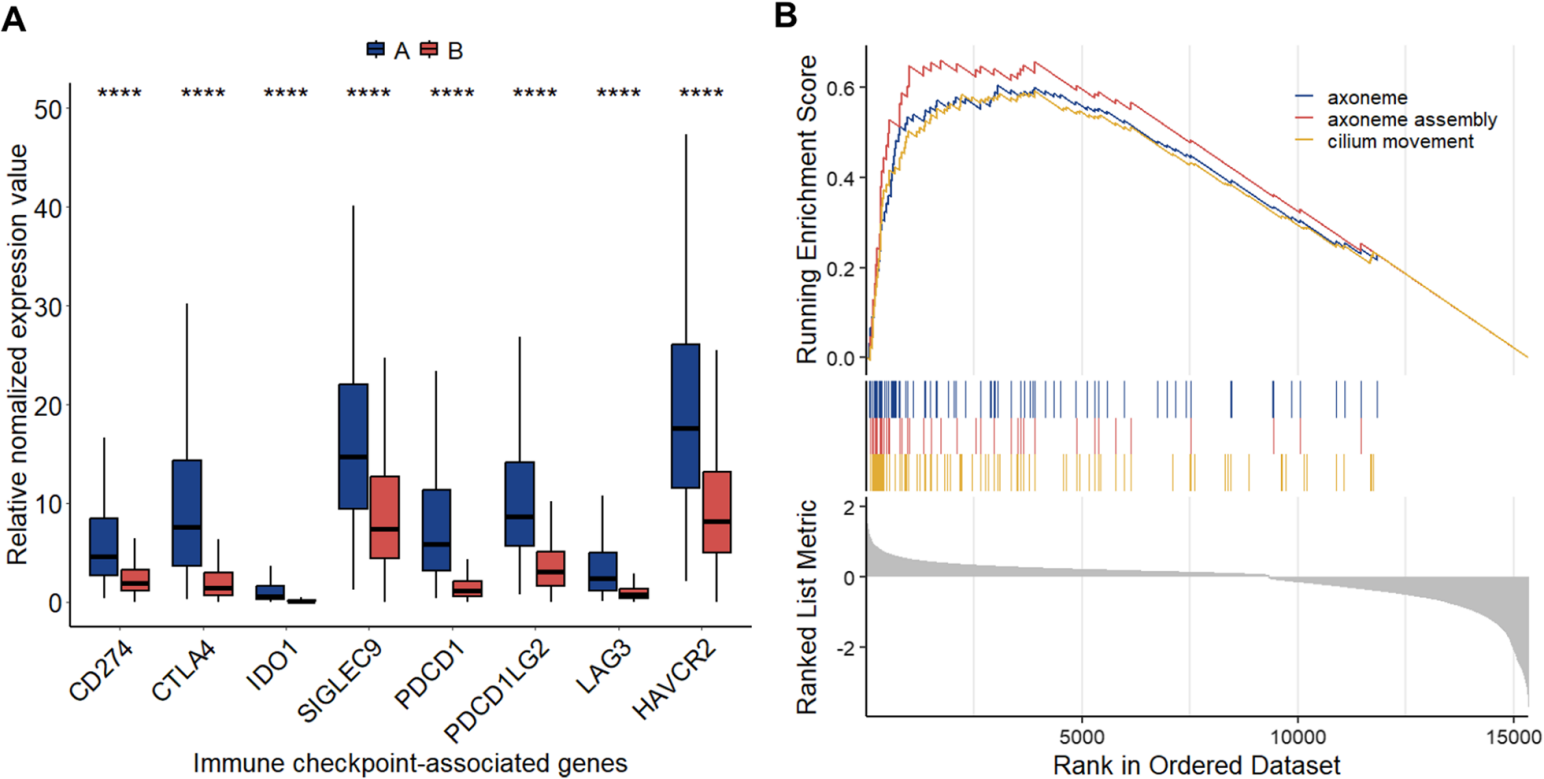
Activation of immune modulators in breast cancer. **A** Expression of immune checkpoint-related genes (CD274, CTLA4, IDO1, SIGLEC-9, PDCD1, PDCD1LG2, LAG3, and HAVCR2) in the Cluster A and B. **B** Enrichment plots showing that Axoneme, axoneme assembly, and cilium movement pathways are significantly upregulated in Cluster A. * ****, *p* < 0.0001

The activation of immune checkpoint molecules in response to immunological stimulation was a potentially significant intrinsic immune escape mechanism. Therefore, we selected *CD274, CTLA4, IDO1, Siglec-9, PDCD1, PDCD1LG2, LAG3,* and *HAVCR2* to evaluate the immune activity of each cluster. Wilcoxon test indicated that all eight immune checkpoint-related genes were substantially over-expressed in the Cluster A (Fig. 5A). Additionally, the GSEA revealed activation of axoneme, axoneme assembly, and cilium movement pathways in Cluster A (Fig. 5B).

### Construction of radiomics signature

**Fig. 6:**
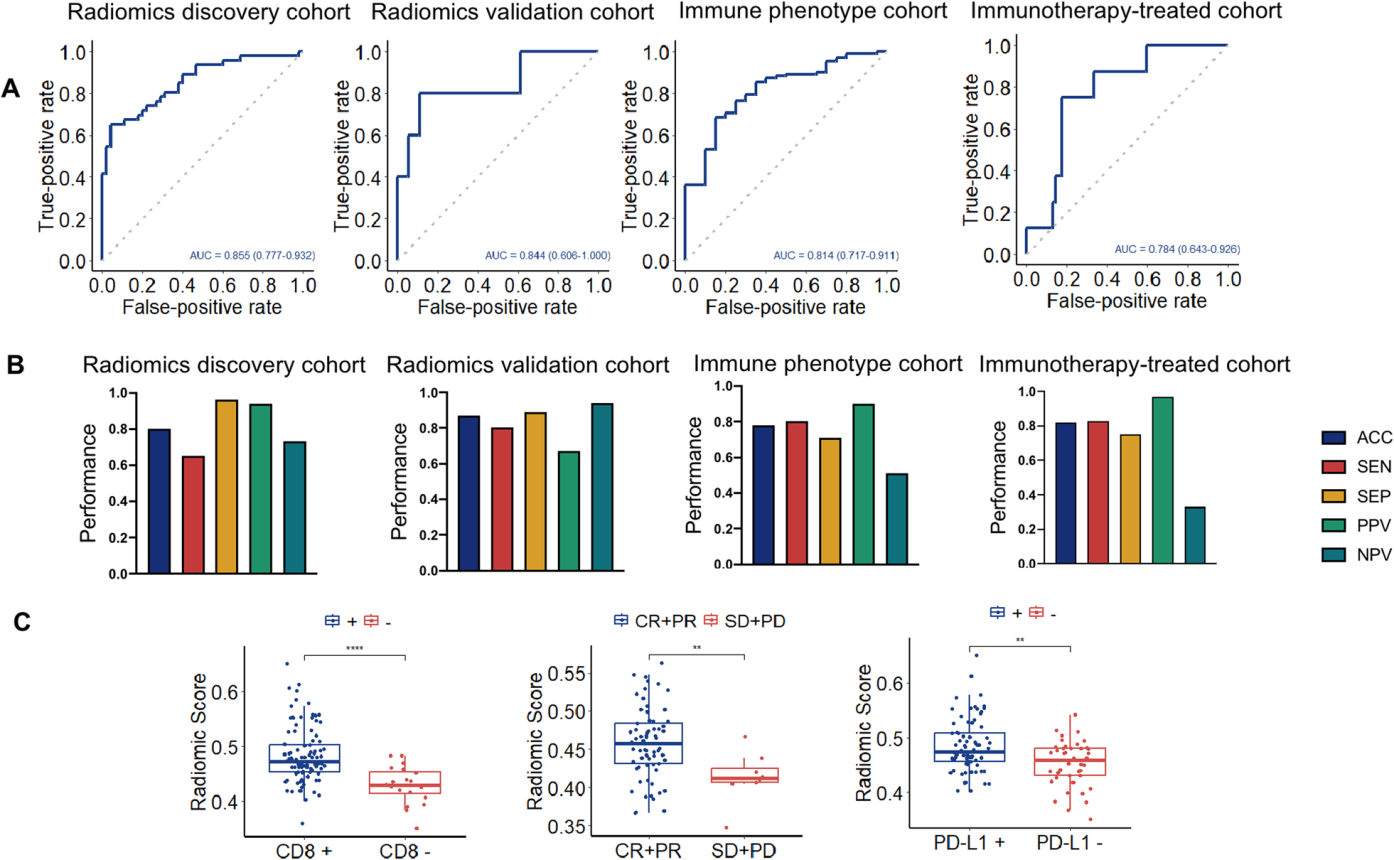
Performance of radiomics signature in the training and validation sets. **A** Diagnostic efficacy of radiomics signature in the training set and three independent validation sets. **B** Evaluation metrics of radiomics signature in the training set and three independent validation sets. **C** Comparison of rad-scores for different immunophenotypes, immunotherapy responses, and PD-L1 expression, respectively. * ****, *p* < 0.0001. **, *p* < 0.01; ACC, accuracy, SEN, sensitivity, SEP, specificity, PPV, positive predictive value, NPV, negative predictive value

The radiomics signature for predicting TME phenotypes was developed using random forest machine learning. **S**pecific details of the top 20 features that contributed the most to the evaluation of feature importance are presented in Table S8. In the Radiomics discovery cohort, the radiomics signature achieved an AUC of 0.855 (95% confidence interval [CI]: 0.777–0.932, *p* < 0.05) for identifying TME phenotypes (Fig. 6A). The optimal cut-off value calculated for the Radiomics discovery cohort, based on the Youden’s index, was 0.528. Using this threshold value, patients' tumors were classified as "hot" or "cold." The confusion matrix was used to further assess the performance of the model, and Figs. 6B display the sensitivity, specificity, and other evaluation metrics, which all suggested that the signature had excellent predictive performance.

### Validation of radiomics signature

The radiomics signature demonstrated a comparable capacity to predict TME phenotypes in the Radiomics validation cohort, achieving an AUC of 0.844 (95% CI 0.606–1.000, *p* < 0.05) (Fig. 6A). When the optimal cut-off point was set at 0.569, the sensitivity and specificity approached 0.80 and 0.89, respectively (Fig. 6B).

The efficacy of the radiomics signature in predicting TME phenotypes in the Immune phenotype cohort revealed an AUC of 0.814 (95% CI 0.717–0.911, *p* < 0.05). (Fig. 6A). The assessment metrics further illustrated the strong prediction ability of radiomics signature (Fig. 6B). Patients in the immune-inflamed (CD8 +) group exhibited substantially higher rad-scores than those in the immune-desert (CD8-) group (*p* < 0.01) (Fig. 6C). Furthermore, the radiomics signature performed well in both HER2( +) and HER2(-)/HR( +) groupings (Additional file 4: Figure S3).

The follow-up period for the Immunotherapy-treated cohort was 6 months following the initiation of immunotherapy. The AUC for the radiomics signature's predictive capacity for objective response (complete and partial response) and no response (stable disease and progressing disease) to immunotherapy was 0.784 (95% CI 0.643–0.926, *p* < 0.01) (Fig. 6A). However, there was no statistical significance (*p* > 0.05) in the capacity of the radiomics signature to identify disease control (stable disease, partial response, and complete response) and progressing disease. Patients with an objective response had higher rad-scores than those with stable disease and progressive disease (*p* < 0.05) (Fig. 6C), whereas patients with disease control did not have higher rad-scores than those with progressive disease (*p* > 0.05).

### Relationship between the radiomics signature and immune checkpoint

Radiomics predictors were able to distinguish between cases with positive (tumor proportion score ≥ 1 +) and negative PD-L1 expression in the Immune phenotype cohort (*p* < 0.01) (Fig. 6C). Specifically, immune infiltration was significantly higher in the PD-L1-positive cases than the PD-L1-negative one. *CD274* expression was greatly enhanced in the immune-inflamed phenotype compared to the immune-desert one, as shown in Fig. 5A. Thus, the consistency of our results demonstrates the superior predictive power of our radiomics signature for the TME phenotypes. However, the radiomics predictor did not demonstrate the capacity (*p* > 0.05) to differentiate between other clinicopathological factors (e.g., TNM stage, breast cancer molecular subtype) (Additional file 5: Figure S4).

### Interpretability of radiomics features

**Fig. 7:**
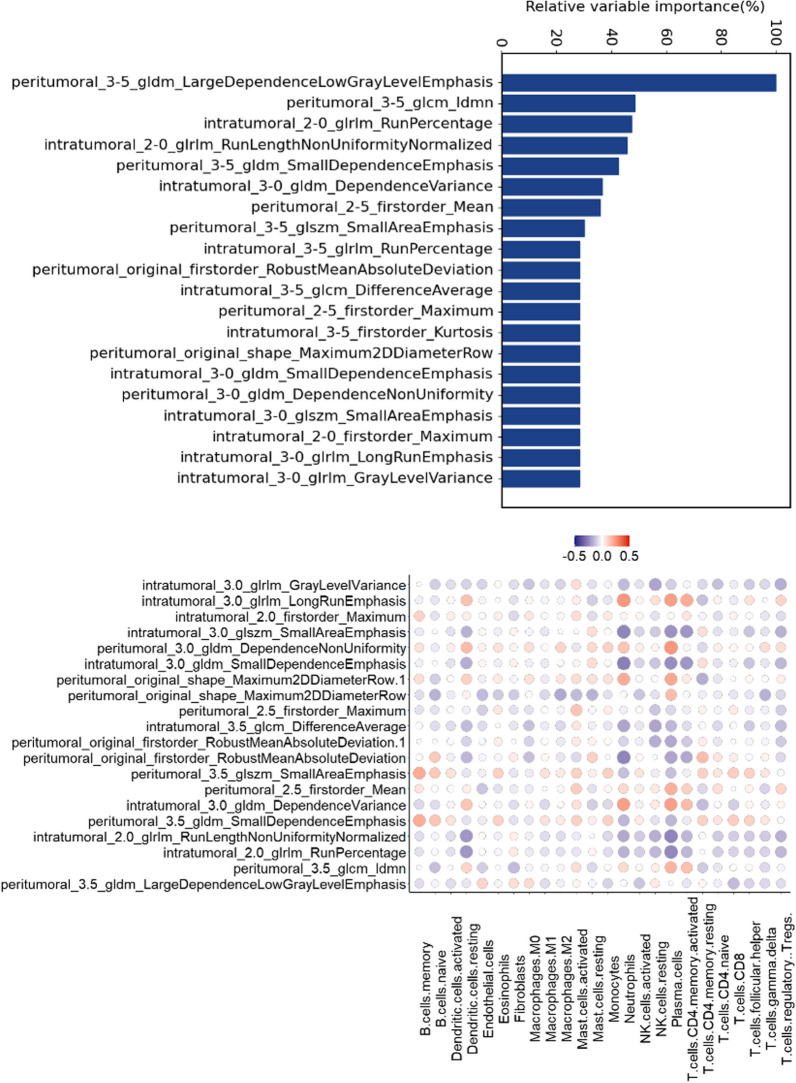
Interpretability of radiomics features. **A** Random Forest for feature importance assessment. **B** Correlation assessment of the top 20 contributing radiomics features with the abundance of 24 TME cell infiltration

The linkage between the top 20 contributing radiomics features and the abundance of 24 immune cell and stromal cell infiltrates was examined in the Radiomics discovery cohort (Fig. 7, Additional file 1: Table S9). The findings revealed a significant link between most cell types and radiomics features (*p* < 0.05), with this correlation being most pronounced and largely negative between Neutrophils, Nk cells, Plasma cells, and T cells CD4 memory activated and radiomics features. Due to similar importance of each radiomics feature, the correlation level with the abundance of 24 cellular subpopulation was evenly distributed.

## Discussion

Our work utilizes extensive TCGA breast cancer data to identify two heterogeneous breast cancer TME subtypes and their related clinical significance. The Cluster A is referred to as a "hot tumor," whereas the Cluster B is categorized as a "cold tumor." We emphasize the remarkable features of clusters that may cause immune escape: enhanced expression of immune checkpoint markers in Cluster A and deficiencies in innate immune cell recruitment in Cluster B. Our current analysis aligns with the findings from Xavier Tekpli and Wen Huang et. [13, 14] and is consistent with the immunologic principles outlined in a previous article [40]. Our findings carry significant implications for clinical translation, particularly in aiding in the identification of individuals who will benefit from ICIs therapy. We have discovered a "hot tumor" cluster in breast cancer, where elevated expression of immune checkpoint markers may contribute to immunological escape. Despite most clinical studies demonstrating a success rate of less than 10% for ICIs in triple-negative breast cancer [6, 24], patients in this category often undergo numerous rounds of immunotherapy without prior assessment of immune checkpoint proteins. Notably, the effectiveness of PD-L1 inhibitors in first-line monotherapy can reach 25% [6], aligning with the proportion of "hot tumors" identified in our current analysis. We anticipate breast cancer cells responsive to ICIs would also respond to chemotherapy. Consequently, ICIs-sensitive cells may be eliminated after several rounds of chemotherapy, impacting the levels of immune checkpoint proteins post-surgery. Given the heightened effectiveness of ICIs over chemotherapy, we suggest the early utilization of ICIs should in the "hot tumor" of breast cancer.

Although immunotherapy is increasingly employed in breast cancer, PD-L1 expression is the most widely used biomarker associated with tumor immune checkpoint treatment; yet, for the majority of tumors, PD-L1 measured by IHC assays are unsatisfactory as a biological marker for the anti-PD-1/PD-L1 therapy [41]. Therefore, new biomarkers for predicting and monitoring patient response to immunotherapy are required, representing an essential step toward the age of precision immuno-oncology. Our work aims to address this need by presenting an MRI-based biomarker that, given the ubiquitous availability and routine use of MRI, could be highly relevant and accessible. We developed a radiomics signature of TME cell infiltration from MR images and explored the relationship between the imaging features, transcriptome data, TME phenotypes, and clinical response to immunotherapy in our research. Furthermore, we confirmed the radiomics signature of the TME phenotypes in three different cohorts, demonstrating its association with the immune phenotypes while predicting clinical outcomes in patients treated with the anti-PD-1/PD-L1. A study published in Lancet Oncol by Roger Sun et al. [33] developed a radiomics signature of tumor immune infiltration from CT scans, which can predict patient immunotherapy performance. In contrast to that study, Roger Sun's study assessed tumor immune infiltration by the abundance of infiltrating CD8 cells. Current articles examining TILs by IHC typically use CD8 + TILs levels to represent TILs [42–44]. However, immune cells located within mesenchyme and cancer nest, calculated by transcriptomic data, include lymphocytes and various phagocytes. This broader calculation of immune cell infiltration is in contrast to TILs within IHC, which generally refer to lymphocytes within the mesenchyme, indicating that the infiltration of immune cells calculated by transcriptome data covers a broader range.

In this work, the radiomics signature mainly comprises textural features that objectively, statistically, and multidimensionally depict tumor biology and its inherent heterogeneity. An direct interpretation of these features is that homogeneous and low-density tumor and peripheral rings are linked with enhanced immune cell infiltration [33]. To further explain the biological aspects of the radiomics features, we correlated the top 20 extracted significant radiomics features with the abundance of 24 TME cell infiltrations. We observed a substantial association between the majority of cell populations and the radiomics features, suggesting that our extracted features may represent the phenotypes of breast cancer TME. Notably, a recent study [33] examined features extracted from the intratumoral region and its peritumoral region when treated by breast cancer immunotherapy, finding a link between immune cell infiltration (CD8) into the tumor and a CT-based radiomics signature, which was consistent with our findings. Furthermore, another study [45] concluded that peritumoral textural features might indicate TME, supporting our findings.

A growing number of studies [19, 46, 47] have explored radiomics as a predictor of immune infiltration or immunological pathways in recent years, although few patients in these research underwent immunotherapy. Another work employed machine learning to predict overall survival and responsiveness to immunotherapy, demonstrating a link between radiomics and genetics or biology in lung cancer, providing a theoretical foundation for future research [47]. Our work adds to this body of evidence on the relationship between MR radiomics, TME phenotypes, and outcomes of anti-PD-1/PD-L treatments.

The immune-inflamed phenotype and the immune-desert phenotype are the only two immune phenotypes that we chose to examine in our research. We made decision so that the rad-score could be classified as high or low, and the findings could be analyzed appropriately. We focused on the degree of immune and stromal cell infiltration in breast cancer TME since a lack of immune infiltration has been linked to poor immunotherapy response [25, 48]. To account for the spatial distribution of immune and stromal cells in the tumor TME, we considered two areas independently: the tumor and peripheral margin. We used the radiomics signature to predict the distinct phenotypes of TME based on imaging features for both areas. As expected, future prospective direction might incorporate these three phenotypes, as well as possible additional immune phenotypes, as we gain a better understanding of breast cancer and its microenvironment immune function [12].

Our research has certain shortcomings. First, there are more than 24 different kinds of stromal cells, making it challenging to cover all phenotypes adequately. To address this, we focus on three kinds of these cell subpopulations—adaptive and activated innate immune cells, inactivated innate immune cells, and non-immune cells—as a partial solution to this issue. Secondly, the heterogeneity of the cohort poses a challenge. We aimed to provide a comprehensive method for characterizing breast cancer that would encompass its underlying behavior by choosing diverse cohorts. We utilized different cohorts for training and assessment to prevent overfitting. Consequently, we attempted to homogenize the data by establishing quality standards, pre-selecting pictures based on the reconstruction algorithm, and considering image acquisition parameters. However, these constraints resulted in a reduced number of eligible patients. Due to the retrospective nature of our work, further validation of the findings necessitates a large prospective study.

## Conclusions

Collectively, our research showcases that the categorization of breast cancer TME phenotypes into two distinct, heterogeneous clusters. Radiomics emerges as a promising, non-invasive, cost-effective, and reliable tool for characterizing TME phenotypes and clinical response to immunotherapy in patients. While the findings necessitate validation in a larger prospective trials, they underscore the potential for developing non-invasive biomarkers in the realm of immunotherapy.

### Supplementary Information

**Additional file 1. **Supplementary Tables.

**Additional file 2: Figure S1.** Determination of the optimal cluster number of breast cancer TME phenotypes. A-D, Silhouette analysis for KMeans clustering on the breast cancer data. E, Nbclust test of the breast cancer data.

**Additional file 3: Figure S2.** The distribution of different clinical factors of Cluster A and B. The distributions of age and molecular type are significantly different between the two clusters, whereas the values of the other clinicopathological features are similar. * ****, *p* < 0.0001; *, *p* < 0.05; NS, no significant.

**Additional file 4: Figure S3.** Performance of radiomics signature in the Immune phenotype cohort. A, Diagnostic efficacy of radiomics signature in different molecular subtypes. B, Evaluation metrics of radiomics signature in different molecular subtypes. * ACC, accuracy; SEN, sensitivity; SEP, specificity; PPV, positive predictive value; NPV, negative predictive value.

**Additional file 5: Figure S4.** The efficiency of radiomics signature for differentiating various TNM stages (left) and molecular subtypes (right).

## Data Availability

The data that support the findings of this study and the computer code are available from the corresponding author upon reasonable request.

## Abbreviations

TME: Tumor microenvironment
PD-L1: Anti-programmed cell death ligand 1
ICIs: Immune checkpoint inhibitors
TCGA: The Cancer Genome Atlas
TCIA: The Cancer Imaging Archive
IHC: Immunohistochemistry
RECIST: Response Evaluation Criteria for Solid Tumors
AUC: Area under the subject operating characteristic curve
LUMP: Leukocytes Unmethylation for Purity
