# Supplementary figures and images for: Development of a machine learning-based radiomics signature for estimating breast cancer TME phenotypes and predicting anti-PD-1/PD-L1 immunotherapy response
Source: Breast Cancer Res. 2024 Jan 29;26:18. doi: 10.1186/s13058-024-01776-y (PMC10823720; doi:10.1186/s13058-024-01776-y)

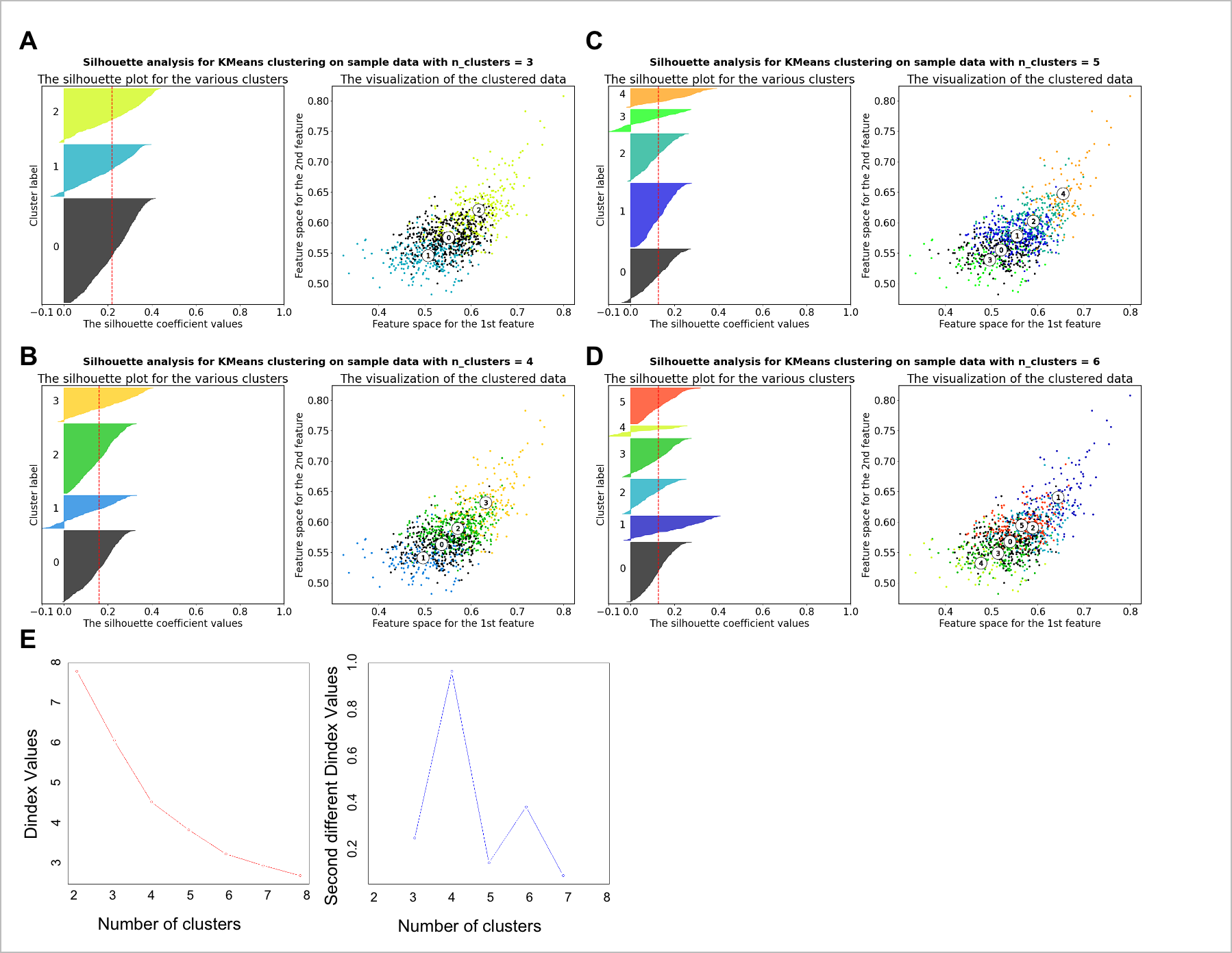

Supplement: Supplementary file 2 — Additional file 2: Figure S1. Determination of the optimal cluster number of breast cancer TME phenotypes. A-D, Silhouette analysis for KMeans clustering on the breast cancer data. E, Nbclust test of the breast cancer data. [file 13058_2024_1776_MOESM2_ESM.tif]

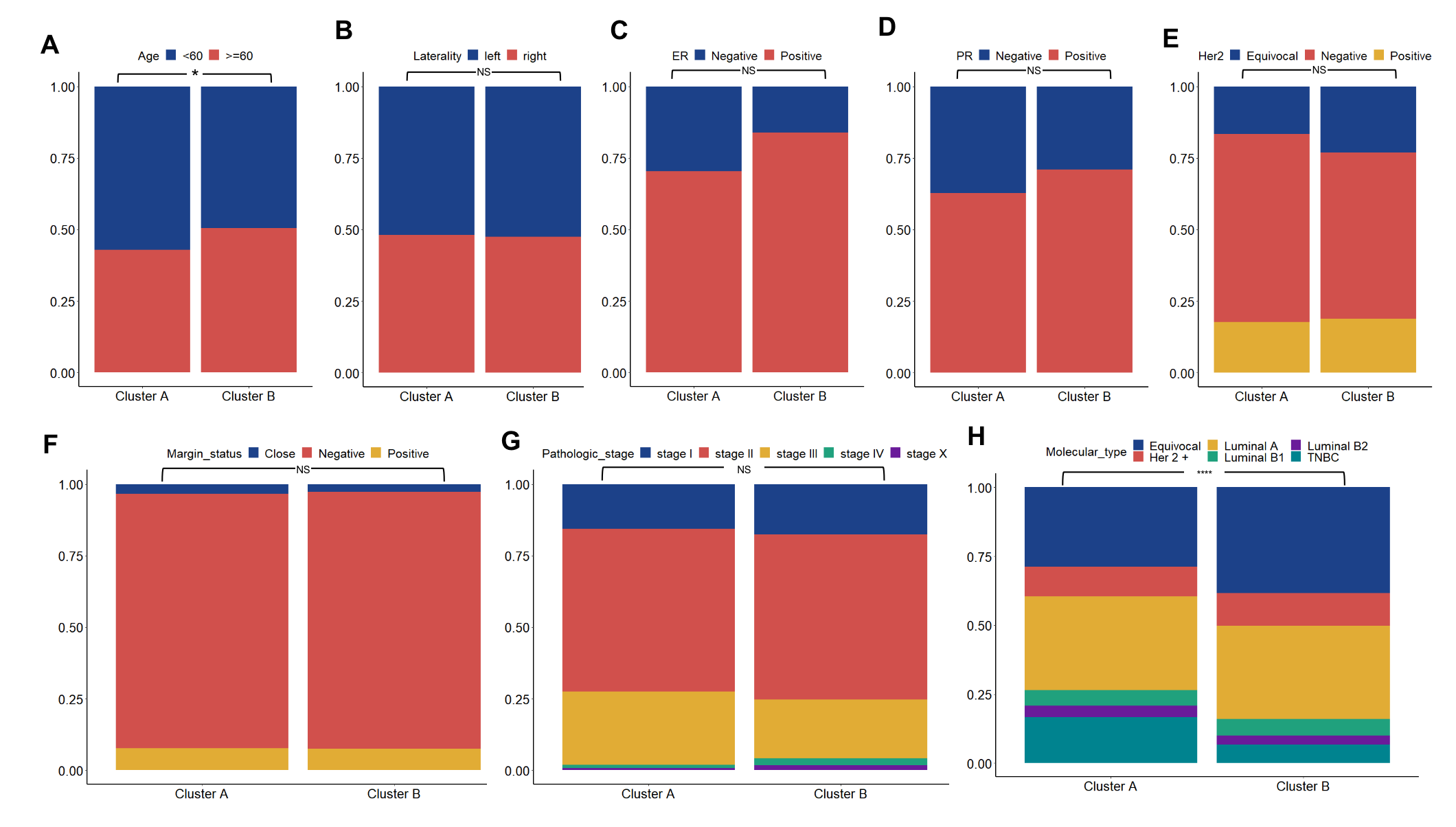

Supplement: Supplementary file 3 — Additional file 3: Figure S2. The distribution of different clinical factors of Cluster A and B. The distributions of age and molecular type are significantly different between the two clusters, whereas the values of the other clinicopathological features are similar. * ****, p < 0.0001; *, p < 0.05; NS, no significant. [file 13058_2024_1776_MOESM3_ESM.tif]

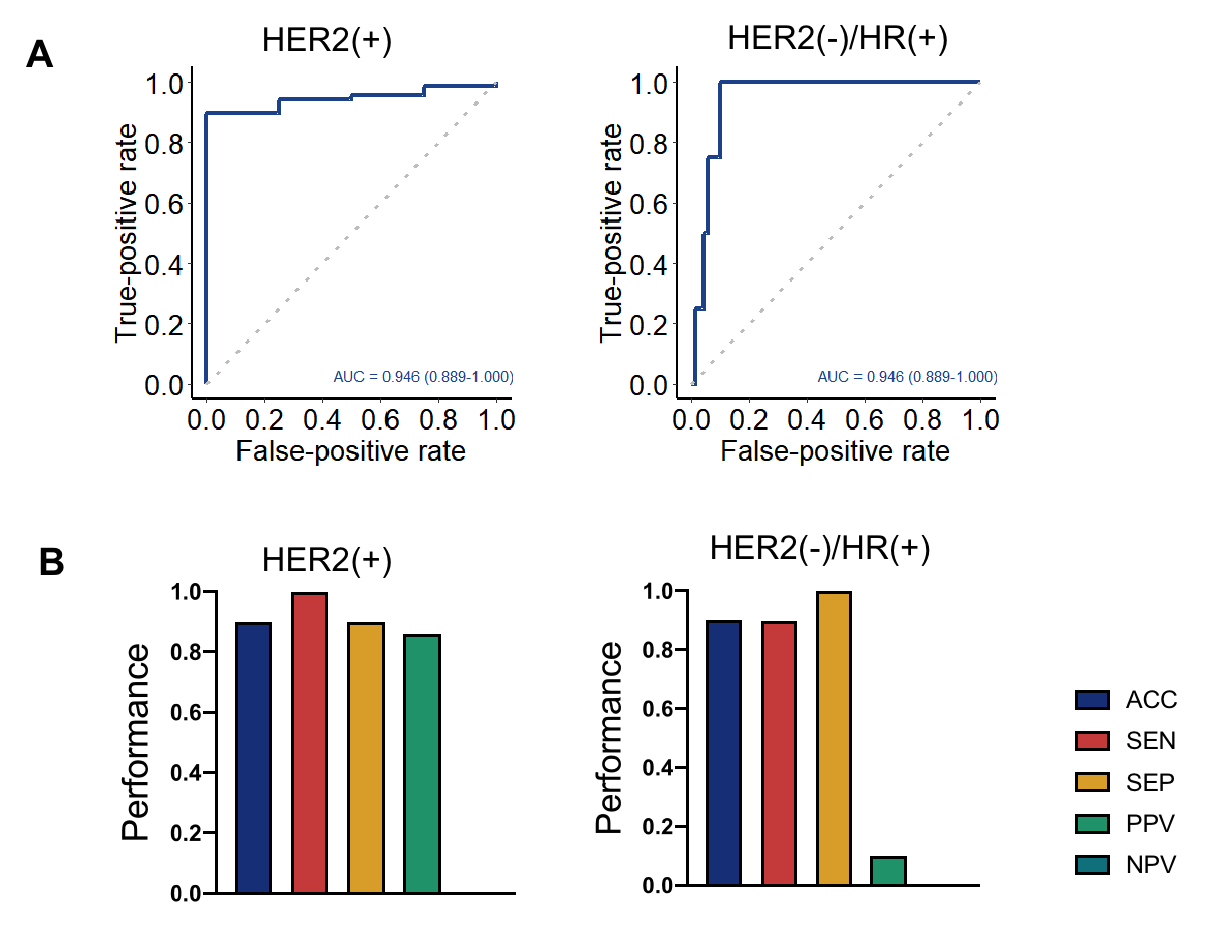

Supplement: Supplementary file 4 — Additional file 4: Figure S3. Performance of radiomics signature in the Immune phenotype cohort. A, Diagnostic efficacy of radiomics signature in different molecular subtypes. B, Evaluation metrics of radiomics signature in different molecular subtypes. * ACC, accuracy; SEN, sensitivity; SEP, specificity; PPV, positive predictive value; NPV, negative predictive value. [file 13058_2024_1776_MOESM4_ESM.tif]

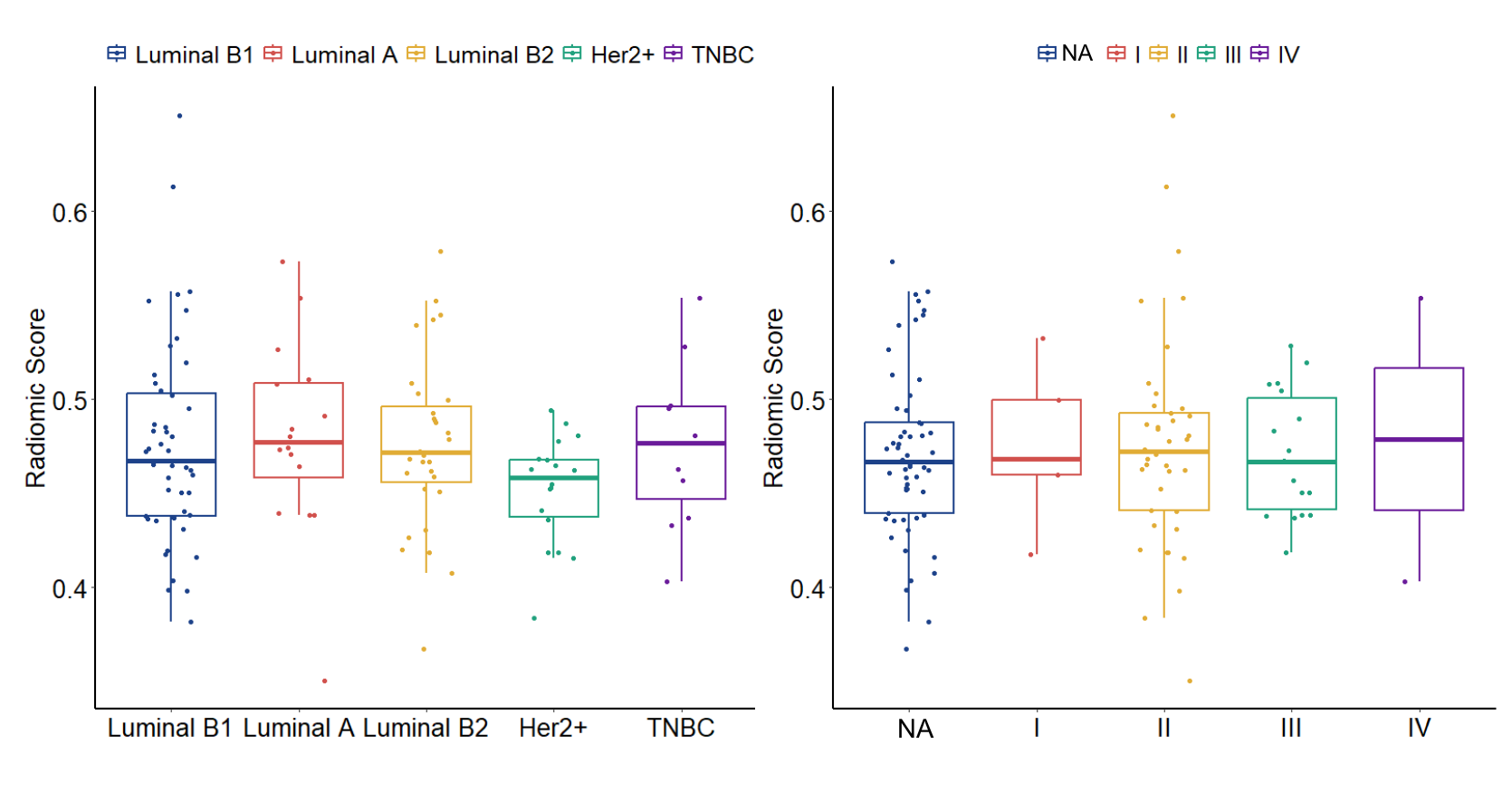

Supplement: Supplementary file 5 — Additional file 5: Figure S4. The efficiency of radiomics signature for differentiating various TNM stages (left) and molecular subtypes (right). [file 13058_2024_1776_MOESM5_ESM.tif]
